# Causes and Consequences of Freezing Cold Injuries in the Norwegian Armed Forces from the Soldier’s Perspective—A Qualitative Study

**DOI:** 10.3390/ijerph23040444

**Published:** 2026-03-31

**Authors:** Tuva Steinberg, Mona Anita Kiil, Arne Johan Norheim, Trine Stub

**Affiliations:** 1 The Norwegian Armed Forces Joint Medical Services, Sessvollmoen Garnison, 2058 Sessvollmoen, Norway; 2 Norway’s National Research Centre in Complementary and Alternative Medicine (NAFKAM), Department of Community Medicine, Faculty of Health Service, UiT The Arctic University of Norway, Hansine Hansens veg 19, 9037 Tromsø, Norway

**Keywords:** freezing cold injuries, military service, soldiers’ perspective, risk factor, long-term sequelae

## Abstract

**Highlights:**

**Public health relevance—How does this work relate to a public health issue?**

- This study enhances our understanding of the causes and consequences of freezing cold injuries (FCI), which significantly impact individuals living and working in cold environments.
- It provides valuable insights into the prevalence and risk factors associated with FCI, contributing to a broader understanding of this public health issue.

**Public health significance—Why is this work of significance to public health?**

- Freezing cold injuries can lead to serious long-term effects on both professional and social life.
- Short- and long-term consequences, such as chronic pain and numbness, may result in an inability to work or reduced capacity to engage in outdoor activities.

**Public health implications—What are the key implications or messages for practitioners, policy makers and/or researchers in public health?**

- The findings of this study can contribute to ongoing efforts to prevent and manage freezing cold injuries, improving current practices and interventions.
- Developing standardized diagnostic criteria for freezing cold injuries is essential and warranted.

**Abstract:**

Freezing cold injury (FCI) is a localized injury caused by prolonged exposure to sub-zero temperatures, posing a significant health risk to military personnel operating in extreme climates. This study aimed to explore how FCI affects the health and lives of soldiers in the Norwegian Armed Forces (NAF), addressing the broader context of its impact on military readiness and the personnel’s well-being. Sixteen participants with a history of grade 2 FCI were recruited through the Norwegian Armed Forces Health Registry and interviewed. Systematic content analysis was used to identify key themes related to the causes and consequences of FCI, as well as the influence of military culture. The findings indicate that FCI results from interacting environmental, behavioural, and systemic factors. Environmental contributors included cold exposure and limited opportunities to eat or drink. Behavioural and individual contributors included reduced awareness of bodily cues in cold conditions and the need to remove gloves for weapon handling under time pressure. Systemic contributors, particularly military cultural norms emphasizing strict discipline and toughness, were perceived to discourage early symptom reporting and the use of preventive measures. Most participants (11/16) reported long-term consequences, including chronic physical discomfort, functional limitations, and negative impacts on their careers and daily lives. This study highlights the significant short- and long-term consequences of FCI, which extend beyond physical harm to affect soldiers’ overall health, functioning, and career progression. The findings emphasize the need for targeted preventive measures to mitigate the risks of FCI and protect military personnel in extreme climates.

## 1. Introduction

Cold weather injuries (CWIs) are a major concern for individuals exposed to extreme climates, particularly in Arctic and sub-Arctic regions [1,2], or operating at high altitudes [3]. These injuries occur when the body is subjected to prolonged exposure to cold, leading to a range of physiological responses that can result in temporary and permanent damage. CWIs are typically categorized into three main types: freezing cold injuries (FCI), non-freezing cold injuries (NFCI), and hypothermia. Each type poses unique challenges, with varying degrees of severity and long-term consequences [4,5,6].

FCI are localized cold-induced injuries that occur when skin and underlying tissues freeze due to exposure to sub-zero temperatures. FCI can lead to cell death, tissue necrosis, and, in some cases, amputation [7,8].

Exposure to cold environments has profound physiological effects, impairing soldiers’ physical health, mobility, cognitive performance, and decision-making under stress [9]. FCI pose immediate risks and carry long-term consequences, potentially undermining operational effectiveness and endangering personnel [10]. Consequently, the Norwegian Armed Forces (NAF) place significant emphasis on the prevention, early identification, and effective management of FCI [11].

The spectrum of FCI outcomes ranges from temporary discomfort to permanent tissue damage and disability, underscoring the critical importance of robust prevention, timely recognition, and comprehensive intervention strategies [6,8,12]. However, FCI are not merely medical conditions. They are deeply embedded within broader cultural, environmental, and operational contexts, particularly in military settings [13]. Moreover, individuals with Raynaud phenomena are at increased risk of FCI [14] and are frequently medically disqualified from military service under standard criteria.

**Figure 1:**
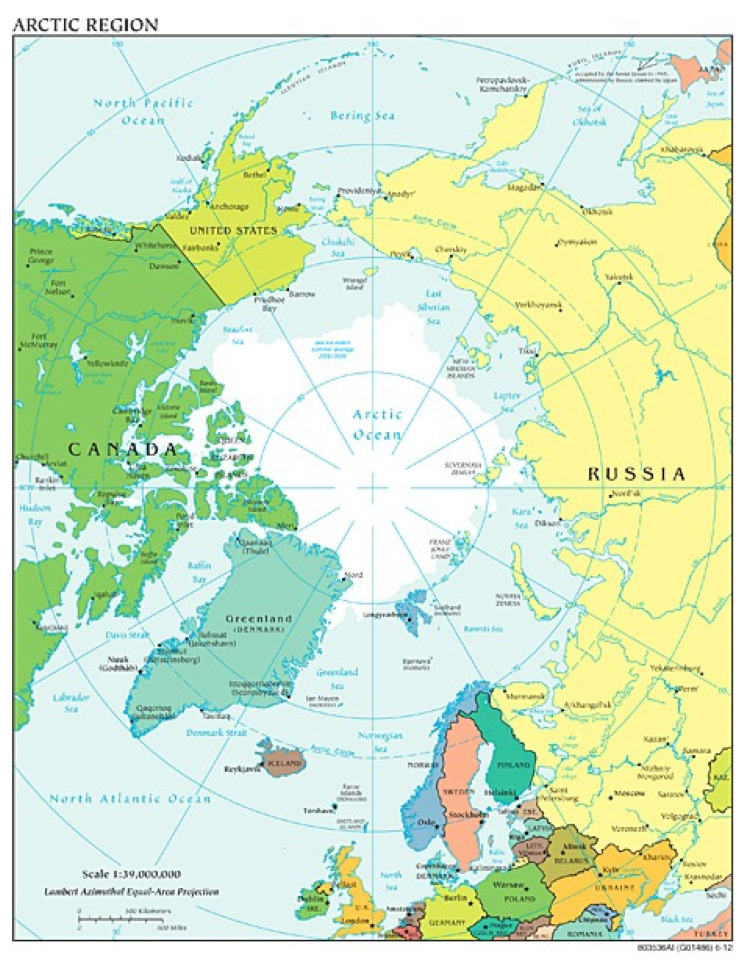
Map of Norway including the area above the Arctic Circle [17].

Given Norway’s geographical positioning (Figure 1) and the harsh climates encountered during military training and deployment scenarios, understanding FCI and implementing preventive measures are essential for the military medical community [2,5,15]. As a member of the North Atlantic Treaty Organization (NATO), Norway holds a strategic role in Arctic and sub-Arctic defence. This responsibility extends beyond protecting national forces. It includes sharing expertise with NATO allies involved in joint exercises and operations in cold environments [9,16]. Enhanced knowledge of FCI prevention and management are essential to maintaining readiness and resilience across the alliance [9].

Norwegians are culturally attuned to winter conditions and outdoor life [18]. A common saying, often attributed to Alfred Wainwright [19] captures this mindset: “There is no such thing as bad weather, only unsuitable clothing”. This cultural outlook fosters a basic understanding and respect for cold weather and an expectation that Norwegians, civilians, and military personnel should be able to handle harsh climates [16]. In a military context, this cultural outlook translates into a mindset of overcoming environmental challenges as a core duty, shaping both training and expectations.

The NAF employs a layered clothing system to reduce the risk of cold-related injuries. Soldiers are trained to adjust their clothing based on activity levels and environmental conditions, minimizing the risks of overheating, perspiration, and subsequent chilling, common precursors to FCI. Standard-issue equipment, including insulated gloves, boots, and headgear, further mitigates heat loss from vulnerable areas. This practice is reinforced through operational training that emphasizes the importance of clothing regulation in dynamic field conditions [11].

The NAF maintains high standards for physical and mental endurance, particularly in cold weather conditions [2]. Soldiers are expected to operate effectively despite environmental stressors, reflecting both practical survival needs and cultural values of resilience and adaptation to nature. Understanding how soldiers perceive their injuries, manage long-term consequences, and navigate challenges in their military careers and daily lives could provide valuable insights for improving prevention and care strategies [20].

While quantitative studies have explored the physiological and epidemiological aspects of these injuries, there is a dearth of qualitative research examining the subjective experiences, adaptations, and long-term consequences faced by the affected soldiers which is needed as qualitative methodology and is well suited to gather such information [21]. Thus, delineating the soldiers’ histories may provide valuable insights into how they perceive their injuries, how they manage long-term effects, and what challenges they face in their military careers and daily lives [1,22].

The primary aim of this qualitative study was to explore soldiers’ perspectives on factors contributing to FCI within military contexts. Specifically, the study sought to: (i) identify the causes and circumstances leading to FCI, (ii) assess the short- and long-term consequences for affected soldiers, and (iii) examine the possible influence of military culture on developing FCI.

## 2. Materials and Methods

### 2.1. Design

This study is based on a qualitative research design consisting of 16 semi-structured interviews with soldiers who sustained a grade 2 FCI during active service in the NAF. A qualitative study focuses on going in-depth and capturing insight to understand complex and subjective phenomena in context [23].

### 2.2. Study Setting

NAF currently employs around 18,000 in active service, including conscripts. Norway has a system of compulsory military service for both men and women, ensuring that a broad segment of the population receives basic military training each year. In 2023, 9138 individuals completed their initial military service. Of these, 3037 were women, corresponding to a share of 33.2 percent [24].

### 2.3. Inclusion and Exclusion Criteria

To be included, participants had to be diagnosed with a grade 2 FCI during military service, be at least 18 years old, and provide informed consent to join the study. Individuals who were misdiagnosed and unable to give informed consent were ineligible for inclusion.

### 2.4. The Semi-Structured Interviews

Semi-structured interviews are a validated method when seeking new and in-depth knowledge about the participants’ experiences [25]. This approach is particularly useful when examining a phenomenon like FCI where there is previously limited knowledge, as it is suitable for generating such information [25]. It may shed light on how cultural and organizational factors influence soldiers’ responses to FCI, offering a more holistic perspective on the issue [23,26]. The interview guide was developed based on findings from a survey from 2023 [5], relevant scientific literature and the research team’s expertise. It was then piloted. The interview guide included questions about *the cause of FCI; consequences of frost damage; external factors/climate; communication/attitudes; clothing and equipment; knowledge and education.* The interview guide is attached as Supplementary Material (File S2).

### 2.5. Recruitment

Potential participants were identified from individuals registered with FCI in the Norwegian Armed Forces Health Registry (NAFHR) between 2004 and 2021 who also took part in a national survey (*n* = 1141) conducted in 2021 where 220 soldiers sustained a grade 2 FCI [5,27]. Among these, 88 soldiers answered that they were asked to participate in an interview study and if interested they contacted the first author by email. Despite two reminder invitations, 16 participants gave their informed consent. Thus, purposive sampling was used in this study [23]. 

### 2.6. The Participants

A total of 16 participants were included in the study, 8 women and 8 men. The participants were between the ages of 23 to 54, with a mean age of 27.1 at the time of their interviews. They all suffered from FCI grade 2, mostly on their fingers, but also on their feet and ears (Table 1). Mean years since injury was 4.5 years.

### 2.7. Data Collection

The interviews were conducted through Teams (a cloud-based video conferencing platform) and audio-recorded with the consent of the participants. Most of the interviews lasted between 30–60 min. The interviews were conducted in Norwegian by the first author (TUS). No one else was present during the interviews. To ensure the anonymity of each participant, an identification number (ID#) was used. Notes were taken during and after the interviews. The interviewer had no previous experience conducting qualitative research. However, she is trained as a medical doctor and familiar with patient consultations and taking the patient’s case histories. During each interview, the first author requested permission to re-contact participants if further clarification was needed, which all participants agreed to. Of the 16 participants, 7 were recontacted for further clarification regarding their injury and possible treatment. The transcripts were not returned to participants for comments or feedback.

### 2.8. Data Analysis

The interviews were transcribed verbatim and translated into English by a professional service. The analysis was conducted using conventional content analysis [28]. The success of content analysis depends on the coding process, and in this study, the codes were coded inductively. All the authors read the transcripts carefully [23]. Then, the first (TUS), the second (MAK), and the third author (TS) read the transcripts several times and created codes based on recurring themes related to the study aims. In this first step, they received input from the research team, and disagreements were discussed until consensus was reached. The data were entered and coded into NVivo 1.61 [29]. After reviewing the coding, the authors discussed all disagreements. A total of 16 codes were organized into 3 main and 4 subthemes due to similar information. Three main themes (including one cross-cutting theme) were identified: Causes of FCI (sub-themes: Parallel events; Management of equipment; Lack of systematic reporting); Consequences of FCI (sub-theme: How FCI influences career, work, and lifestyle); The military culture. The consolidated criteria for reporting qualitative studies [30] were followed to ensure the methodological quality of the study.

The trustworthiness of the findings was supported by having a rich variation in the participants’ perspectives. The dependability of the analysis and findings was addressed by having a consistent interview guide for all the participants [21].

Sample sizes for qualitative studies are typically not predetermined, but as a team we agreed that after 14 interviews, no new information emerged. However, two additional interviews were conducted to ensure that saturation was achieved [31,32]. Credibility was achieved by having regular meetings to discuss the findings, in addition to several independent coders during the data analysis process [23]. We drew on the participants’ verbatim quotes and were reflexive about how our role, and assumptions may have influenced the interpretation of the data for confirmability [21] especially since the interviewer is an employed doctor in the NAF. Concerning the transferability of the findings, we included representation from several types of military personnel and their various perspectives.

### 2.9. Ethical Considerations

The study was approved by the Regional Ethical Committee (REC) with the identification number 278429. Before completing the interviews, the researcher informed the participants about the aim of the study, the purpose and content of the interview. Written and verbal informed consent was obtained from the participants.

As described above, this article adheres to the COREQ (Consolidated Criteria for Reporting Qualitative Research) checklist [33] to ensure transparency and rigour in its methodology and reporting. The COREQ checklist is available in supplementary materials (File S1).

## 3. Results

FCI is the standardized medical terminology for frostbite. In the NAF, the reported annual incidence is approximately 120–150 cases. For clarity and consistency, we will employ the term FCI throughout this section, acknowledging that participants in the interviews used a variety of alternative expressions to describe the condition.

### 3.1. Causes of FCI

FCI is primarily a physical condition, yet the participants highlighted how its occurrence was influenced by a range of interconnected factors beyond mere exposure to cold temperatures.

#### 3.1.1. Parallel Events

The participants’ experiences highlighted how FCI arises from a complex interplay of parallel events, inadequate or missing equipment, the necessity of precise equipment handling, and intense time constraints, as described by one participant (ID2). She developed FCI during a three-day ski march under extreme conditions. On the final day, temperatures dropped to −30 °C as the group descended a steep hill, where they stood in line and pushed sleds. Despite reporting numbness in her fingers and toes to the officer, the response was to continue moving. Upon returning to base, she was diagnosed with FCI at the military medical ward.

Participant (ID15) described sustaining FCI from parallel events. While awaiting a COVID-19 test, he was separated from his troop and required to stand outdoors in freezing temperatures during a severe storm. He explained: *I was in isolation due to suspected COVID-19 infection and had a fever when I was required to wait outside during a storm, leading to FCI on all my fingers.* Despite wearing the warmest gloves available, prolonged exposure led to numbness in his fingertips, which he initially dismissed due to lack of knowledge about FCI. Over the following days, he remained isolated while awaiting the test results, but the numbness in his fingers persisted. Upon reporting his symptoms to a medical professional, FCI was confirmed.

Four (of 16) participants (ID2, ID3, ID6, and ID15) identified fatigue and dehydration as critical parallel events exacerbating the risk of FCI.

Participant (ID2) described a challenging winter ski march during a prolonged exercise, where exhaustion, thirst, hunger, and freezing temperatures took a significant toll. She recalled how the combination of fatigue and extreme cold made it nearly impossible to stay hydrated. She said: *We were so tired and dehydrated that even melting snow for drinking water became a challenge. I think that the combination of these events made me vulnerable for getting FCI on my fingers*.

Thinking back on an experience during a winter exercise where temperatures dropped to −37 °C, (ID6) explained: *We didn’t receive a mid-layer jacket as part of our gear. Instead, we had to use layers of multiple 200-g and 600-g garments under our jackets, which wasn’t nearly as warm as the proper jacket would have been.* He described the challenges during the final days of a seven-day exercise, particularly while standing guard for two hours at a time and running outdoors with limited food. He noted: *The exposure to low temperatures leaves you so cold and unfocused that, over time, you stop noticing or caring for yourself*.

#### 3.1.2. Management of Equipment

Tasks requiring precise manual dexterity frequently led soldiers to remove protective gloves, directly increasing their risk of FCI, as explained by participant (ID4): *The officers tell you never to remove gloves, yet equipment handling often requires precise actions impossible with bulky mittens.* Participants (ID2, ID5, ID9, ID11, ID13) all cited weapon handling as tasks necessitating glove removal.

Participant (ID14) further described how standard protective measures practiced during recruit training, such as operating weapons with mittens, often diminished after an initial rehearsal period. Thus, this lack of sufficient training increased the risk in later field exercises, as described: *During recruit training, we practiced handling weapons with mittens extensively, but this practice decreased during subsequent duty.*

Ill-fitting or defective equipment heightened vulnerability to FCI. Examples included missing gaiters, oversized gloves, and insufficiently warm jackets. Participant (ID5) recounted how poorly sized gloves forced her to remove protective gear in extremely cold conditions. *The equipment didn’t fit, so I had to remove my gloves to put up the tent. This caused my fingers to get stuck on the tent buttons, leading to FCI*, she explained.

Participant (ID4) recalled the seventh day of an exercise in −40 °C. To stay warm, they kept moving throughout the day, but when he finally removed his gloves in the tent, he discovered blisters on his fingers. Reflecting on this, he said: *I hadn’t noticed anything before that, so it’s hard for me to say when or where it happened, but it was discovered in the tent*. Participant (ID8) described a winter exercise characterized by urgency: *Although training emphasized keeping mittens on, pressure to perform tasks quickly forced me to remove gloves briefly, leading to FCI.* Another soldier (ID1) acknowledged removing gloves under intense pressure, despite awareness of safety guidelines: *I knew removing gloves was against guidelines, but tight time constraints made me take risks to complete the task.*

Participant (ID14) described overlooking his own condition while checking recruits in −20 °C, he neglected to check himself. After returning indoors, he noticed white spots on his fingers, that developed into blisters. Reflecting on the experience, he said: *It must have been at least an hour before I could warm them (fingers) up, and maybe a couple of hours after that the blisters appeared.*

#### 3.1.3. Lack of Systematic Reporting

According to the participants in this study, systematic reporting gaps led to inconsistent tracking and management of FCI, as described by participant (ID4) who noted: *Even after receiving treatment, there was no clear follow-up or documentation of my injury.* Similarly, participant (ID8) specifically points out the absence of formalized reviews and documentation following cold-weather exercises. He commented: *We never had formal reviews or documentation after our cold-weather exercises. This meant no lessons were learned officially.*

Participant (ID6) initially received no formal documentation or report after her injury. She was instructed by her senior officer simply to deal with it herself, which, according to her, delayed the official intervention.

On the other hand, some participants had positive experiences when reporting FCI. Participant (ID13) described their officers as highly attentive and supportive, taking cold-related concerns seriously. They ensured that the soldiers warmed up in vehicles if needed and acted immediately if signs of FCI were observed. The participant appreciated their proactive approach, acknowledging that lack of own actions contributed to the situation.

Participant (ID14) described his experience with leaders as generally positive, noting that his leaders were knowledgeable and experienced in handling FCI. Communication with his immediate superior was effective, and the concerns were taken seriously, saying: *It was my leader that encouraged me to file a report and ensured appropriate steps were taken to address the situation.*

### 3.2. Consequences of FCI

This section describes how the FCI affected the injured soldiers’ lives even years after their military service.

#### How FCI Influences Career, Work and Lifestyle

For 11 (of 16) participants, the FCI had long-term consequences. However, 5 of 16 experienced full recovery (ID10, ID11, ID12, ID14, ID16), as illustrated by participant (ID10) who noted: *I had numbness in my feet for some years, but recently it has diminished. I will say that I now have regained normal sensations.* In line with this, participant (ID11) contracted FCI after touching the helmet button, which caused temporary pain, but the pain healed without lasting effects. In addition, participant (ID14) experienced that the FCI healed quickly, leaving no long-lasting effect. He has been out in cold weather without feeling any pain.

Participant (ID2) still has numbness in her fingers five years after the FCI. Although the sensation has improved, she still finds herself quickly affected by cold weather, necessitating the constant use of gloves, except in the summer. This situation has led her to pursue indoor work, as outdoor jobs are impossible due to the injury. Reflecting on this, she says: *Straight after military service, I chose to work indoors as a computer scientist. Due to my cold and sensitive fingers, I cannot work outdoors.*

In line with this, participant (ID6) struggled with tingling, numbness, and painful hands six years after his injury. The symptoms increase, particularly during temperature fluctuations. At first in the interview, he downplays his discomfort but eventually admits that it is bothersome. He noted: *I don’t find that typing on a keyboard makes a difference, but I’m sitting here with my legs crossed, one hand between my thighs, and the other hand typing is cold.*

Participant (ID15) explained that he was discharged from the military service due to FCI. However, he also suffered from meniscus injury in the knee. Both events led to this discharge. Looking back, he said: *It was frustrating because I wanted to be there* (in the military). *I had joined with several friends, shared a room with them, and knew many others from before.* He was very disappointed about the discharge because he had hoped to build a career in the Armed Forces.

Participant (ID11) experienced FCI on his left index finger during service, which he believed was minor. Although the injury caused some discomfort, such as blistering and temporary pain, he was initially deemed fit for duty and allowed to continue participating in activities. However, shortly after, a doctor abruptly decided he was unfit for service, leading to his unexpected discharge. Reflecting on this, he said: *It was irritating. Very irritating. That’s why I applied to get back in.* Despite the discharge, he successfully navigated the complaint process, obtained a medical certificate, and re-entered service.

Participant (ID3) shared that the injury had altered her habits in cold weather. While her daily life remained largely unaffected, she took precautions when she wanted to grab cold items, using gloves as a protective measure. If she forgot her gloves, she pulled the sleeves of her sweater down to cover her fingers. *In this way I try to protect my fingers*, she said.

Moreover, participant (ID5) still experienced severe pain two years after her FCI, which has affected her habits. She planned her outdoor activities (cross-country skiing) carefully. She said: *I cannot freeze my hands at any time, because my fingers get very, very sore, when exposed to cold weather.*

Another participant (ID13) noted that she got pain when preparing cold food, such as preparing chicken for dinner. *I get pain and numbness in my fingers*, she explained. Another participant (ID7) has experienced numbness and pain in fingers every winter since the injury five years ago, although it is not as intense as right after the injury. She explained: *I always wear gloves when I remove ice from the car window.*

Participant (ID16) experienced more annoyance from the FCI. She said: *It was a bit painful in the beginning, some tingling and such, but that’s gone. But now I’m sitting in my car outside the office, and now my feet are freezing so I need to take a short walk to get them warm again.*

### 3.3. Cross-Cutting Theme

An overarching theme emerged from the other two themes and interviews in which the participants described their experiences and reflections on how strict discipline, while sometimes necessary, contributed to the risk of FCI. This cross-cutting theme was identified as the military culture.

### 3.4. The Military Culture

The soldiers, who had sustained FCI, described cultural values in the military such as strict discipline, hierarchy and toughness as factors that might influence the soldiers’ risk of getting FCI.

Soldiers encountered the challenge of navigating competing priorities: fulfilling mission objectives and adhering to orders on the one hand, while attempting to prioritize their own well-being and protect their health on the other. Rigid hierarchical power structures sometimes reinforced this tension, which complicated open communication and self-advocacy (more difficult).

Due to military culture such as toughness and risk-taking, discussing challenges or discomfort was perceived as a sign of weakness according to the participants in this study.

Participant (ID7) recounted an accident she experienced shortly before a winter exercise, which resulted in an elbow injury. Due to this injury, she was unable to assume the required ready position (plank) during rehearsals. However, this information (the injured elbow) was not communicated to all officers. She recalled an incident where, despite her injured elbow, she was ordered to assume the plank position: *I was strictly told to go down, down. So, I didn’t dare do anything else. I stood there right on the ice without gloves, in the plank position. That’s probably when I got it (the FCI). We stood there for quite a while, probably two minutes.*

A soldier (ID9) experienced FCI during a training exercise due to strict adherence to orders. The unit was a close-knit group, with soldiers emphasizing uniformity in actions. During one particularly cold evening, with temperatures dropping to 15 °C below zero and strong winds, a fellow soldier forgot his hat. As a result, the entire group was ordered to remove their hats, resulting in FCI on his ear. Reflecting on the experience, he expressed regret for not refusing the order, acknowledging that his compliance stemmed from a fear of appearing weak in his own eyes. He emphasized the importance of officers prioritizing soldiers’ long-term well-being. *I believe if someone does something that can negatively affect a soldier’s future health, it is a hindrance to positive development.*

This sentiment aligns with that of participant (ID8). He recalled being rushed while handling his weapon and regretted not taking the time to keep his gloves on. *I should have disobeyed orders. Even though there was time pressure, and I was told to hurry, I should have taken more time and kept the gloves on.* However, he admitted that fear of reprimand prevented him from speaking out.

Participant (ID6) described an incident where a junior officer dismissed his concerns after he reported FCI. The officer merely instructed him to *swing his arms and keep moving.* However, the participant questioned the junior officer’s ability to accurately assess the situation, noting that the officer was only a year older than himself. It was not until a superior officer intervened that he received the appropriate attention. Reflecting on the experience, he remarked: *I kind of experienced it as a failure of care, though that might be a strange term to use for a soldier, poor follow-up, though!*

Participant (ID4) described feeling embarrassed when reporting FCI to his superior. He explained that the underlying expectation was for the company to demonstrate capability and toughness. These expectations led to a sense of weakness when a soldier got FCI. He reflected: *Getting an injury such as FCI was seen as underperformance. I believe this pressure to meet high standards may have discouraged some individuals from voicing their concerns.*

Similarly, participant (ID9) shared his experience of feeling socially stigmatized because he had to always wear a beanie (after FCI on his ear), which visibly set him apart from others who wore berets. He explained: *Due to this, I think I was viewed as a weaker person.*

Participant (ID5) highlighted the absence of a culture that encouraged soldiers to speak up. She remarked: *There was no culture for saying anything. I felt like we were just supposed to endure the cold, so I didn’t think much more about it.* Participant (ID7) also chose not to report his FCI, fearing it would reflect poorly on him. He explained: *I didn’t want to appear as the weak link in my unit, so I said nothing.*

Participant (ID16) described facing an ethical dilemma when deciding whether to speak up about concerns during training. While feeling a sense of loyalty to the system and its methods, she also believed that some practices pushed participants too far and did not align with proper guidelines. Balancing her responsibility to advocate for her team’s well-being with the fear of appearing disloyal created a challenging situation. She ultimately voiced her concerns, particularly about teammates struggling to care for themselves, but found it difficult to reconcile her role as both a participant and someone with leadership experience.

The ethical dilemma of unequal power dynamics was also evident in participant (ID7)’s experience. She described how inconsistent communication and decisions from leaders regarding her medical needs created frustration and uncertainty. Despite receiving a medical exemption from a civilian doctor, a military leader overruled it, requiring her to participate in a strenuous exercise with limited accommodations. Reflecting on this, many participants observed that the military could have provided clearer guidelines and support mechanisms to mitigate injuries.

## 4. Discussion

This study demonstrates that rather than being solely a consequence of extreme cold, FCI reflects a complex interplay of environmental stressors, physical demands, and organizational dynamics. Understanding these interactions is essential to prevent injury and mitigate long-term consequences for health and operational readiness.

### 4.1. Causes of FCI

Our findings indicate that prolonged physical exertion, when coupled with limited hydration and reduced nutritional intake, may increase susceptibility to FCI. This aligns with the RTG HFM-310 final report [13]. According to the report, optimal performance in Arctic conditions rests on a comprehensive foundation. It requires appropriate cold-weather clothing and equipment, as well as environmental knowledge built through education and training. It also depends on adequate nutrition and hydration and the body’s responses to cold. Psychological resilience and healthy sleep are additional pillars. Without this integrated preparation, performance declines and vulnerability to FCI increases [13]. Modern Antarctic expeditions align with these findings. Participants use advanced preventive strategies such as advanced clothing, hand and foot warmers, adequate nutrition and hydration and regular monitoring for early signs of FCI. These measures reduce the incidence of cold-related injuries [34]. By contrast, in the NAF, where conscript service is mandatory, many individuals have limited cold-weather experience and less targeted preparation. This may increase risk and contribute to a higher incidence of FCI.

The management and adequacy of equipment emerged as recurring themes in the participants’ accounts, revealing critical gaps in protective measures. According to the participants in our study, tasks requiring fine motor skills (dexterity), such as weapon handling or tent assembly, often necessitated the removal of gloves, directly increasing the risk of FCI. Dexterity can be highly affected by cold environments, and research demonstrates that low environmental temperatures lead to reduced muscle contraction force and manual dexterity. This happens already at a finger temperature of around 14–15 °C [35,36,37] which creates a paradox: soldiers remove gloves to improve dexterity but end up exposed to both reduced dexterity and risk of FCI.

In a study by Steinberg et al. [5] the importance of adequate clothing and equipment in mitigating FCI is further emphasized as 22.3% of the participants attributed FCI to the incorrect use of equipment, and 21.2% cited equipment failure as a contributing factor. Mekjavic et al. [9] identified three key factors influencing FCI risk: environmental conditions, personal readiness, and clothing.

Similarly, in their analysis, Potter et al. [38] used predictive physiological models, which highlighted the inadequacies of clothing and equipment among Russian soldiers in Ukraine. These inadequacies not only compromised the soldiers’ ability to stay warm but also hindered their performance during operations. The participants in our study, also highlights the critical role of systematic reporting and leadership in managing FCI. This is in line with Sullivan-Kwantes et al. [1] in their research on human performance in extreme cold environments, emphasized the necessity of strong leadership to meet operational demands in arctic conditions. They emphasized that formalized and prompt reporting mechanisms are essential to prevent severe injuries, ensuring that emerging issues are addressed before they escalate.

### 4.2. How FCI Influences Career, Work, and Lifestyle

While some participants in our study reported full recovery, the majority experienced persistent symptoms that continued to significantly affect their work, lifestyle, and physical activities, even years after the initial injury. These findings align with a previous Norwegian study, which identified chronic pain, cold hypersensitivity, reduced sensory function and dexterity, and vasospastic disorders as the most frequent sequelae of FCI [39]. Additionally, a Swedish study [40] observed that abnormal thermal and vibration perception thresholds in participants with grade 1 and 2 FCI could persist for months. This aligns with Steinberg et al. [5] reporting that 72% of the participants experienced long-term sequelae from FCI (63% grade 1 and 28% grade 2), respectively.

The lack of standardized criteria established guidelines, and official recommendations for FCI diagnosis [41] challenges the accuracy of diagnoses reported in our study. Diagnostic conclusions often rely on the subjective judgement of attending physicians at local military wards, leading to inconsistencies. Additionally, soldiers who sustain FCI during conscription are excluded from military service, limiting their career prospects. They are also ineligible for compensation, despite estimates from the Norwegian governmental injury compensation system indicating that FCI results in a 5–20% lifelong disability [42].

These factors, lack of proper diagnosis, exclusion from military service, and absence of financial support, may contribute to underreporting of FCI [2], exacerbating the burden of long-term health complaints observed in our study.

As shown in our study, the long-term consequences of FCI are not confined to professional settings. They extend into the realm of daily life, where they shape how individuals manage routine activities and influence their overall quality of life [43]. Long-term sequelae of chronic conditions, such as FCI, can significantly alter daily habits and impose lifestyle adjustments. According to the participants in our study, these adjustments include a preference for indoor work, the use of gloves to protect cold-sensitive hands from discomfort, the need for precautions when handling cold or frozen food, and the careful planning of outdoor activities to avoid pain.

Such strategies reflect broader coping mechanisms, which are critical for maintaining functionality and well-being in the face of chronic conditions. Coping, defined as the ability to navigate life’s challenges while preserving mental and emotional health [44], is particularly relevant for individuals with FCI. With a mean age for FCI in NAF of 20.5 years [5], these findings highlight the significant burden of FCI on young individuals in physically demanding professions, such as the military. Similar to young adults with chronic illnesses, soldiers with FCI often strive to maintain a sense of normalcy and avoid being perceived as weak or burdensome [45]. This often leads to the concealment of symptoms, as participants sought to overcome their injuries to remain fully integrated within their group. Downplaying symptoms has been noticed both among active-duty service members and veterans who are bothered by the “suck it up and drive on” mentality [46]. This mindset does not arise in isolation. It is deeply embedded in military culture. The expectation to endure hardship, obey orders, and place mission above self is integral to soldier identity and group cohesion. While such values are essential for operational effectiveness, they also create an environment where health complaints may be silenced or minimized.

For military personnel, exposure to physical and psychological stressors poses significant risks to both health and performance. The development of effective coping skills to manage these military-related stressors is strongly linked to enhanced life satisfaction and resilience [47]. Moreover, coping strategies are known to predict variance in resilience, underscoring their importance in fostering adaptability and mental strength [48]. We believe this reflects a key quality of the participants in our study, demonstrated by their effort to minimize the impact of their injuries and their willingness to continue training and overall service in the NAF. Participants described various strategies they employed to adapt to their injuries, such as wearing additional layers of clothing to stay warm, using different clothing to protect affected areas, and adjusting their outdoor activity to avoid pain or discomfort. However, this might not always be applicable with military standards of clothing.

Many participants in the study also expressed a strong desire to avoid exclusion from their teams, fearing stigmatization as the “weakest link.” For example, one participant, sent home due to FCI on a finger, actively sought to return to service, motivated by a deep sense of belonging and camaraderie with peers. Another participant, who sustained FCI on his ear, struggled with the visibility of his injury, as it required him to wear different headgear, making him stand out. Over time, such pressures can erode self-esteem and foster feelings of inadequacy, compounding the psychological burden of FCI [49].

These findings align with attachment theory, which suggests that a sense of belonging and connection to others has a calming and protective effect on individuals [50]. In military contexts, this sense of belonging is particularly critical, as it supports both psychological resilience and group cohesion [51]. The concept of belonging to a group is consistent with the literature on attachment, which suggests that the presence of others has a calming effect on individuals [50]. Moreover, a study conducted among 247 college students found that belonging predicted better health perceptions for women and fewer physical symptoms for men. This suggests that a sense of connection to a group is a key support component for the physical health of college students [52].

### 4.3. Military Culture

The findings from this study suggest that military culture plays a complex role in the development and management of FCI. Military culture encompasses both structural factors, such as formal hierarchies and discipline, and normative expectations, such as toughness and endurance. Structural necessities like weapon-handling protocols, high operational tempo, and training schedules can increase cold exposure and reduce opportunities to rewarm. Normative expectations, stoicism, fear of reprimand, and a strong collective orientation can suppress self-protective actions and delay help-seeking. The interplay of these factors shapes soldiers’ responses to physical strain, potentially leading to health risks. While some structural necessities are indispensable for operational effectiveness, they may also create situations in which health concerns are subordinated to mission demands [53].

Dziak [54] notes that the military culture functions as a distinct social system, structured by discipline, hierarchy, and rigorous training. These elements are critical for operating under high-stress conditions but also distinguish the military from civilian life, where norms emphasize individual rights and autonomy. As described by [ID8], taking off his gloves when handling the weapon in a stressful situation resulted in FCI on his fingers. He did not speak up for himself for fear of reprimands. In the military, values such as obedience and collective performance are more strongly internalized compared to civilian society. For many Norwegian conscripts, the transition into this system may be particularly abrupt, as they move from a democratic upbringing focused on rights and individual expression into a structure where autonomy is limited and group priorities dominate [55].

Over the last years, research on cold exposure and adaptation has advanced considerably, providing new insights into both physiological responses and strategies for injury prevention [56]. It indicates that short-term repeated cold air exposure—even over several consecutive days—does not significantly improve protective physiological adaptations such as skin blood flow, thermal comfort, or manual dexterity [56]. These findings suggest that brief cold acclimatization protocols may be insufficient to enhance resilience against FCI risk, underscoring the importance of longer-term strategies and comprehensive preventive measures. In practice, this means that military training environments cannot rely solely on limited pre-exposure sessions to induce effective adaptation. Instead, prevention should emphasize appropriate clothing systems, proactive monitoring, early recognition of cold injury symptoms, and individual education. Incorporating these insights into training doctrine could help reduce the burden of cold-related injuries while acknowledging the physiological limits of short-term adaptation [13].

However, technical knowledge alone is not enough. Its effectiveness depends on leadership decisions and the cultural framework within which soldiers operate. When norms of endurance or strict adherence to mission objectives outweigh health considerations, available knowledge may remain underutilized [57], as illustrated by (ID7), who was commanded to stand in the ready position in ice water despite a documented injury in her shoulder. This situation illustrates that pushing soldiers beyond proper guidelines can have serious consequences, leading to ethical dilemmas for officers. Participant (ID16) described the challenge of balancing her responsibility to advocate for soldiers with her loyalty to the system.

While endurance and discipline are essential for operational readiness, they must be balanced against another core military value, the commander’s responsibility to safeguard the health of subordinates [11]. This responsibility is particularly important in peacetime and during conscription, when young soldiers perform compulsory service and cannot be expected to accept self-destructive risks. The persistence of 120–150 new FCI cases annually in Norway, despite preventive guidelines such as the Handbook in Winter Service [11] highlights an opportunity to strengthen implementation and explore innovative approaches to reduce the incidence of FCI. Refining training practices, enhancing equipment use, and reinforcing a culture of early reporting, the NAF can further reduce the burden of cold injuries while maintaining operational effectiveness.

### 4.4. Strengths and Limitations of the Study

This study should be interpreted with consideration of its strengths and limitations. While qualitative analysis offers valuable insights into how participants understand and interpret situations, it cannot be used to establish causal associations [58]. The study involved interviews with sixteen soldiers who had experienced FCI. Although conducting more than sixteen interviews might have provided a broader range of experiences, no significant new insights emerged during the final two interviews. This led to the conclusion that the information power was sufficient, and additional interviews would not have substantially altered the findings [32]. Furthermore, the interviews were in-depth, yielding rich and detailed material. Participants also exhibited notable similarities in their personal histories, concerns, and coping strategies.

Another strength of this study is the geographic diversity of the participants, who were drawn from military bases across Norway. However, the study is based on data from a specific group of soldiers diagnosed with FCI grade 2 and diagnosis were verified during interviews. Soldiers with FCI grades 1 or 3, as well as those who have not reported their injuries, may have different experiences. As a result, the findings may not fully represent the experiences of all soldiers with FCI in Norway. The study included participants registered in the NAFHR with grade 2 FCI. Diagnosis was verified during interviews.

A limitation of the study is that the interviewer and first author is a physician at the NAF, which may have influenced the participants’ responses. Participants may have provided responses they perceived as favourable to the interviewer (please the researcher bias [25]). Alternatively, the interviewer’s role as a physician in the military may have fostered trust, encouraging participants to share accurate and detailed information. To minimize potential hierarchical challenges and reduce the influence of military rank or authority, she chose not to wear a military uniform during the interviews. However, this was accounted for and balanced by the rest of the research team, who are not affiliated with the NAF and consisted of one individual with an MD/PhD, one anthropologist (PhD), and one who holds a PhD in medical science. This collaboration contributed ensure an objective and thorough analysis of the data.

Another limitation was that recruiting participants from the NAFHR, focusing solely on grade 2 FCI, may introduce selection bias, potentially underrepresenting soldiers with milder or more severe FCI or those who did not report FCI. We focused on grade 2 FCI due to its clear diagnostic features (notably clear-fluid blisters), while grade 1 is more challenging to diagnose with certainty. While this approach may have limited generalizability, it ensured a more reliable identification of cases. Moreover, the sample comprised 8 men and 8 women. This differs from the gender distribution in the NAF, where approximately 32% of conscripts are women [24]. Consequently, it is uncertain whether a higher proportion of women would have yielded different findings. Despite two reminder invitations, no additional participants were recruited. Moreover, saturation was achieved after 14 interviews, meaning that no additional information was received (see Section 2.8 Data analysis) [23].

### 4.5. Practical Implications

The findings of this study on FCI have clear and immediate practical applications. They can seamlessly integrate into ongoing efforts to prevent and manage FCI, enhance and improve existing practices. Prevention efforts regarding FCI should consider the cultural dynamics within the military by focusing on actions, such as promoting systematic and early reporting processes for FCI, developing standardized diagnostic criteria for FCI to ensure consistency across the NAF and prioritizing the health, safety, and operational readiness of soldiers. Moreover, (encouraging) a shift in cultural attitudes within the military should be encouraged to create an environment where soldiers feel empowered to report FCI without fear of stigma or being perceived as a weak link in their unit. While protective clothing and equipment are essential, this study emphasizes that the preventive effectiveness depends not only on availability but also on proper use, especially under operational stress, rapidly changing weather conditions, and during military maneuvers (e.g., handling of weapons). Training programmes should focus on the practical application of prevention strategies, such as adjusting clothing during physically demanding tasks. First aid for FCI, involves rapidly rewarming the affected tissue [8]. In practice, this can be difficult during CWOs because rewarming should not be attempted if there is a risk of refreezing, which is common in remote CWOs where garrisons may be far away. Accordingly, emphasis should be placed on preventive measures.

## 5. Conclusions

Beyond the immediate physical harm, the findings underscore the significant short- and long-term consequences of FCI, which extend to soldiers’ careers, daily functioning, and overall health. Furthermore, aspects of military culture, such as strict discipline, hierarchical structures, and entrenched norms, may increase risk for FCI.

From a military perspective, this study contributes to a broader understanding of causes and consequences of FCI, which may have impact on the military strike force and combat endurance when operating in extreme weather conditions in the high north.

## Figures and Tables

**Table 1:** Demographic data of the participants.

Participants	Gender	Age	Location	FCI *	Years Since FCI (4.5 Mean Years)	Conscript **	Employed
ID1	Male	23	West	One finger, one hand	4	X	
ID2	Female	24	North	Several fingers, both hands	5	X	
ID3	Female	22	South	All fingers, both hands	4	X	
ID4	Male	24	North	Two fingers, one hand	5	X	
ID5	Female	54	South	All fingers, both hands	2		X
ID6	Male	25	South	Several fingers, both hands	6	X	
ID7	Female	24	West	Both hands	5	X	
ID8	Female	26	North	Several fingers, both hands	7	X	
ID9	Male	28	South	One earlobe	6	X	
ID10	Male	23	West	Two toes, both feet	4	X	
ID11	Male	24	North	One finger, one hand	5	X	
ID12	Female	24	North	One toe, one foot	3	X	
ID13	Female	23	West	Several fingers, both hands	4	X	
ID14	Male	23	North	One finger, one hand	4	X	
ID15	Male	23	North	Several fingers both hands	4	X	
ID16	Female	44	South	Several toes, both feet	4		X

* FCI location data were obtained from interviews and may vary in accuracy across participants. ** An individual who is compulsorily enlisted into military service under national conscription laws.

## Data Availability

The dataset underlying this paper has not been deposited in any specific repository. However, all datasets and materials used in this study are available upon reasonable request from the corresponding author. Interested parties seeking access to the data must be willing to comply with Norwegian privacy regulations, ensuring adherence to stringent data protection standards.

## Supplementary Materials

The following supporting information can be downloaded at: https://www.mdpi.com/article/10.3390/ijerph23040444/s1. File S1: COREQ checklist; File S2: Interview Guide—Study 2. Reference [59] is cited in the Supplementary Materials.

## Abbreviations

The following abbreviations are used in this manuscript:

NAFKAM	National Research Center of Complementary and Alternative Medicine
NA	Not Applicable
NATO	North Atlantic Treaty Organization
NAF	Norwegian Armed Forces
NAFHR	Norwegian Armed Forces Health Registry
UiT	The Arctic University of Norway
