# Supplementary material for: Causes and Consequences of Freezing Cold Injuries in the Norwegian Armed Forces from the Soldier’s Perspective—A Qualitative Study
Source: Int J Environ Res Public Health. 2026 Mar 31;23(4):444. doi: 10.3390/ijerph23040444 (PMC13116088; doi:10.3390/ijerph23040444)
Supplement: Supplementary file 1 [file ijerph-23-00444-s001.zip › ijerph-3997575-supplementary/ijerph-3997575-supplementary-2.pdf]

Interview Guide – Study 2

Introductory Questions:

- Can you provide your age, gender, and place of residence?
- How much experience do you have in the Armed Forces?
- Where did you serve during your time in the Armed Forces?
- What type of service do you/did you have in the Armed Forces?
- Are you still working in the Armed Forces?
- Did you have any prior experience or knowledge about frostbite?

Main Questions:

- Would you like to share what happened when you got frostbite?
- How has it affected you afterward?
- What do you think was the cause of your frostbite during your time in the Armed Forces?
- How does this injury affect you today?
- What do you think you could have done to prevent your frostbite?
- Looking back now, is there anything you would have done differently?
- Do you have any thoughts on what the Armed Forces could have done differently?
- What would you say to others who want to prevent frostbite?

Closing Question:

- Is there anything else you would like to share or add?
